# “Existential Vacuum” and Axiological Conflict as Correlates of Cognitive–Affective Dissociation in Medical Staff Attitudes Toward Oncofertility in the Pediatric Population—A Preliminary Report

**DOI:** 10.3390/healthcare14101288

**Published:** 2026-05-09

**Authors:** Piotr Pawłowski, Gabriela Orzechowska, Szymon Niedźwiedź, Jakub Dąbrowski, Otylia Kościołek, Natalia Zaj, Małgorzata Mitura-Lesiuk, Aneta Kościołek, Julia Kołodrubiec, Łukasz Młynarczyk, Adrianna Mulewska, Marzena Samardakiewicz

**Affiliations:** 1 Department of Psychology, Psychosocial Aspects of Medicine, Medical University of Lublin, 20-093 Lublin, Poland; marzena.samardakiewicz@umlub.edu.pl; 2 Institute of Medical Sciences, University of Applied Sciences in Chełm, 22-100 Chełm, Poland; aneta.kosciolek@umlub.edu.pl; 3 Student Scientific Club at the Academic Psychological Testing Laboratory, Medical University of Lublin, 20-093 Lublin, Poland; 63050@umlub.edu.pl (G.O.); 63062@umlub.edu.pl (J.D.); 4 Student Scientific Club at the Department of Psychology, Faculty of Medicine, Medical University of Lublin, 20-093 Lublin, Poland; 67872@umlub.edu.pl; 5 Department of Emergency Medicine, University Children’s Hospital in Lublin, 20-093 Lublin, Poland; 6 Department of Pediatric Hematology, Oncology and Transplantology, Medical University of Lublin, 20-093 Lublin, Poland; malgorzata.mitura-lesiuk@umlub.edu.pl; 7 Department of Nursing Development, Faculty of Health Sciences, Medical University, 20-081 Lublin, Poland; 8 Department of Pediatrics, Oncology and Hematology, Medical University of Łódź, 91-738 Łódź, Poland; 9 Department of Pediatric Oncology, Hematology and Transplantology, Poznań University of Medical Sciences, 60-572 Poznań, Poland; 10 Department of Pediatrics, Hematology and Oncology, Medical University of Gdańsk, 80-211 Gdańsk, Poland

**Keywords:** oncofertility, pediatric, attitudes, medical staff

## Abstract

**Background**: Contemporary pediatric oncology confronts medical staff with challenges that are not only clinical but also ethical and existential in nature. The aim of this study was to identify the cognitive and affective factors associated with medical professionals’ attitudes toward fertility preservation procedures (oncofertility) in pediatric patients. In particular, the association of “existential vacuum” (lack of life goals, sense of meaninglessness), value systems, and religiosity on the level of competence and emotional acceptance of these procedures was examined. **Methods**: A cross-sectional observational study was conducted between January and September 2024 in pediatric oncology centers in Poland (Gdańsk, Lublin, Łódź, and Poznań). The study group consisted of 62 medical professionals (62.9% physicians and 37.1% nurses) selected using purposive sampling. The research protocol included an Author-Designed Questionnaire, the Scheler Value Scale (SVS), the Life Attitude Profile—Revised (LAP-R), and the Centrality of Religiosity Scale (CRS-15). Statistical analyses comprised Pearson’s r correlations, multiple regression analysis, and cluster analysis using the k-means method. **Results**: Participants demonstrated a moderate level of substantive competence in oncofertility (M = 2.31 on a 5-point scale). Regression analysis revealed that “existential vacuum” was the strongest negative predictor of competence (B = −0.34; *p* = 0.001), which was found to be a significant negative correlate of professional development in this area. In the affective domain, a pronounced normative conflict was observed: religiosity was negatively correlated with emotional acceptance of the procedures (r = −0.42; *p* < 0.001), indicating tension between medical imperatives and worldview-based beliefs. At the same time, the regression model showed that internalized religiosity and moral values might theoretically function as an “axiological buffer”; however, due to the severe psychometric limitations of the emotional acceptance measure (α = 0.268), these affective associations are highly tentative and unstable. Alternative measurement strategies are required to validate this hypothesis. Exploratory cluster analysis suggested the potential existence of two professional profiles: “Axiologically Integrated” staff members and a larger group of “Existential Skeptics”, who exhibited higher “existential vacuum” and lower psychosocial resources. **Conclusions**: Viewed through a dual-process interpretative lens, a theoretical phenomenon of cognitive–affective dissociation was explored. The highly tentative data suggest that “existential vacuum” might represent a hypothesized barrier to competence acquisition. Furthermore, findings regarding the affective domain—limited by the low reliability of the emotional measure—suggest religiosity could act as a potential source of normative tension. These exploratory profiles serve as hypotheses for future intervention designs rather than definitive clinical mechanisms.

## 1. Introduction

The dynamic development of medicine has led to a significant increase in survival rates among children and adolescents diagnosed with cancer [1]. Currently, cure rates reach as high as 80–90%. However, this success has simultaneously increased the importance of long-term consequences of intensive anticancer treatment, among which one of the most critical is the loss or risk of loss of fertility [2]. For many patients, this prospect may be as traumatic as the cancer diagnosis itself [3].

In response to these challenges, oncofertility has emerged as a relatively new, interdisciplinary field of medicine integrating oncology and reproductive medicine [4,5]. It was developed to address the conflict between the necessity of initiating effective anticancer therapy and the need to preserve patients’ reproductive potential [5]. This discipline also addresses the psychological burden experienced by both female and male patients who were unable to preserve their fertility prior to undergoing gonadotoxic treatment regimens [1,3,6,7]. Current research unequivocally recommends early discussion with patients regarding the risk of infertility, available fertility preservation options, and timely referral for specialized consultations [2,3,8].

Despite the existence of numerous clinical guidelines [9], scientific reports indicate a substantial inconsistency between recommendations and everyday clinical practice. Discussions regarding fertility preservation are not standardized; they are often conducted selectively, and their scope and quality depend on individual staff engagement [3]. In some cases, patients are not informed at all about the availability of such procedures. Consequently, a significant proportion of patients lack sufficient knowledge about the impact of oncological treatment on their future fertility [2,7,8,10].

Previous studies have primarily focused on the level of knowledge among medical staff, the frequency of fertility-related discussions, and organizational or systemic barriers [2,4,5,8,9,11,12,13,14,15,16]. Considerably less attention has been paid to the psychological and axiological determinants of medical staff attitudes toward oncofertility, such as the role of religiosity, value systems, or a sense of meaning in life. Yet these factors may be significantly associated with the interpretation of medical guidelines, clinical decision-making, and emotional comfort during discussions with patients.

The psychological complexity of these clinical decisions can be framed within the Theory of Planned Behavior (TPB), which posits that an individual’s behavioral intentions are shaped by their attitudes, subjective norms, and perceived behavioral control. In the context of pediatric oncofertility, subjective norms often involve a tension between professional medical standards and the perceived disapproval of religious communities. When healthcare providers feel unable to act according to their professional knowledge due to such internal or external constraints, they may experience moral distress. This state occurs when one recognizes the ethically appropriate action but is hindered by institutional barriers or axiological conflicts. Integrating these frameworks allows for a deeper understanding of how an “existential vacuum” (lack of life goals, sense of meaninglessness) might disrupt the alignment between clinical competence and emotional acceptance, potentially leading to the observed “cognitive-affective dissociation” (a theoretical descriptor for the observed divergence between high procedural knowledge and low emotional acceptance resulting from moral distress) [17,18]. To better understand these dynamics, the current study conceptualizes medical staff attitudes through the lens of value-based decision-making and dual-process models, where clinical behaviors are shaped by both cognitive evaluations (procedural competence) and affective responses (emotional acceptance) [19]. Within this framework, the concept of “cognitive-affective dissociation” was introduced for the purposes of this study. It is important to note that this dissociation is not measured as a discrete empirical variable in this study; rather, it serves as a theoretical descriptor for the observed divergence between high procedural knowledge and low acceptance, often resulting from moral distress.

The aim of the present study is to analyze medical staff attitudes toward oncofertility procedures, taking into account demographic variables as well as selected psychological and axiological factors. Particular emphasis is placed on the role of religiosity, preferred values, and existential attitudes as potential correlates of competence and emotional acceptance of fertility preservation procedures. This approach allows for a more in-depth understanding of the mechanisms underlying difficulties in implementing oncofertility in everyday clinical practice and provides a foundation for designing more effective educational and systemic interventions. While an extensive body of literature already addresses clinicians’ beliefs, emotional discomfort, and organizational barriers in oncofertility and end-of-life care contexts, the specific interplay between profound existential constructs, such as “existential vacuum”, and axiological conflicts remains insufficiently explored in pediatric oncofertility. By shifting the focus from strictly logistical barriers to underlying meaning-making processes, this preliminary report aims to provide a nuanced perspective on the psychological mechanisms driving clinical decision-making in this highly sensitive niche. While recent research on fertility preservation exists, it remains predominantly confined to adult oncology settings. By exploring these specific psychological constructs, this preliminary report aims to expand the current understanding of pediatric oncofertility, offering insights into a relatively under-researched niche without overstating its novelty relative to the broader psychosocial literature.

## 2. Materials and Methods

### 2.1. Study Design

The study was observational and cross-sectional in nature and was conducted using a diagnostic survey model. The research project was carried out between January and September 2024. Data collection took place in selected pediatric oncology clinical centers in Poland (Gdańsk, Lublin, Łódź, and Poznań). The study protocol was approved by the Bioethics Committee of the Medical University of Lublin (approval no. KE-0254/12/01/2023), confirming compliance with the principles of Good Clinical Practice (GCP) and the Declaration of Helsinki (WMA Declaration of Helsinki).

### 2.2. Participants and Sampling

It is crucial to emphasize the highly specialized and restricted nature of the target population. Pediatric oncology and hematology care in Poland is heavily centralized, comprising only 17 specialized centers nationwide and a total workforce of approximately 180 actively practicing pediatric oncologists. Consequently, identifying and recruiting a large cohort is inherently constrained by the objective size of the medical workforce in this specific niche [20].

A purposive sampling strategy was applied. The study invited representatives of medical professions who, in their clinical practice, have direct contact with oncology patients of developmental and reproductive age.

Inclusion criteria:

1. Practicing a medical profession: physician (specialist or physician in specialty training) or nurse.
2. Active professional employment in healthcare facilities involved in the diagnosis or treatment of oncology patients (hospital wards, specialist outpatient clinics).
3. Provision of informed, voluntary consent to participate in the study.
4. Complete completion of the research instruments (no systematic missing data in key scales).

Exclusion criteria:

1. Lack of direct clinical contact with oncology patients (e.g., exclusively administrative or laboratory personnel).
2. Professional experience shorter than 6 months (adaptation period).
3. Withdrawal of consent at any stage of the study.
4. Incomplete questionnaire data (more than 5% missing data within a scale).

### 2.3. Research Procedure

The study was conducted using a traditional paper-and-pencil interview method, ensuring full anonymity of respondents. Questionnaire packages were distributed directly at participants’ workplaces (physicians’ and nurses’ duty rooms) in order to increase the response rate and provide comfortable conditions for completing the survey at a convenient time, without time pressure, thereby minimizing fatigue-related bias. Each questionnaire package included written instructions and information regarding anonymity and the scientific purpose of the study. The average time required to complete the full set of instruments was approximately 20–30 min.

### 2.4. Research Instruments

The study employed a battery of four instruments (one author-designed questionnaire and three standardized psychometric scales).

### 2.5. Author-Designed Questionnaire

Developed specifically for the purposes of this study. It consisted of two sections:

(1) A demographic section including age, gender, years of professional experience, education level, and religious affiliation.
(2) A substantive section containing items assessing knowledge of oncofertility procedures (e.g., cryopreservation of sperm, oocytes, and ovarian tissue), attitudes toward their use in minors, and emotional attitudes toward these procedures. A 5-point Likert scale was applied. To establish content and face validity, the newly developed instrument underwent a rigorous heuristic evaluation by an interdisciplinary expert panel, comprising a clinical psychologist and a pediatric oncologist. The panel evaluated item relevance, terminological precision, and clinical applicability, ensuring that the construct representation aligns with the specific operational context of pediatric oncofertility.

### 2.6. Scheler Value Scale (SVS)

The assessment of axiological preferences was conducted using the Scheler Value Scale (SVS) in the Polish adaptation by Piotr Brzozowski (1997) [21]. This instrument, developed on the basis of Max Scheler’s phenomenological concept of values, consists of 50 statements (value items). The respondent’s task is to rate each of the 50 items on a scale ranging from 0 to 100, where 0 indicates that a given value is completely unimportant to the respondent, 100 indicates that the value is of the highest importance. This methodology allows for a precise determination of the subjective significance of individual values in a person’s life and for establishing their individual value hierarchy. The obtained results are grouped into six dimensions (subscales), arranged in a hierarchical order (from lower to higher values): Hedonic values (e.g., pleasure, comfort); Vital values (e.g., physical fitness, health); Aesthetic values (e.g., beauty, elegance); Truth-related values (e.g., knowledge, objectivity); Moral values (e.g., kindness, honor, integrity); Sacred values (both in religious and secular aspects) [21].

### 2.7. Life Attitude Profile—Revised (LAP-R)

Polish adaptation by R. Klamut [22], referred to as the Life Attitude Questionnaire. The scale assesses existential attitudes and sense of meaning in life. This instrument constitutes an operationalization of Viktor Frankl’s logotherapy and enables a multidimensional analysis of the structure of meaning in life, taking into account both its cognitive and motivational aspects. The questionnaire consists of a series of statements to which the respondent indicates their level of agreement using a 7-point Likert scale (where 1 means “strongly disagree” and 7 means “strongly agree”). The following subscales were used in the present study: “Existential Vacuum” and Purpose Seeking [22].

### 2.8. Centrality of Religiosity Scale (C-15)

Polish adaptation by B. Zarzycka (2020) [23]. The scale measures the centrality of religious constructs within an individual’s personality structure. Religiosity is assessed as a continuous variable, incorporating intellectual, ideological, and experiential dimensions. Respondents rate the statements on a 5-point scale (scores from 1 to 5), indicating the frequency of specific behaviors (from “never” to “very often”) or the intensity of beliefs and experiences (from “not at all” to “very strongly”). The overall score, calculated as the mean or sum of points, allows not only for the assessment of the level of religiosity [23].

### 2.9. Statistical Analysis

The collected empirical material was subjected to substantive and logical verification and subsequently coded into a database. Statistical analyses were performed using IBM SPSS Statistics, version 29.0.

Group characteristics and variables were described using frequencies (N) and percentages (%) for qualitative variables, as well as means (M), standard deviations (SD), and medians (Me) for quantitative variables. Internal consistency of the scales was assessed using Cronbach’s alpha coefficient. Relationships between variables (age, religiosity, knowledge, and attitudes) were examined using Pearson’s correlation coefficient (r). Differences in knowledge levels and attitudes depending on demographic characteristics (e.g., religious affiliation) were analyzed using one-way analysis of variance (ANOVA).

To identify determinants of healthcare professionals’ attitudes, multiple linear regression analysis using the ordinary least squares method was conducted. Variables that demonstrated statistically significant associations with the dependent variable in correlation analyses were entered into the regression models. Prior to conducting the multiple linear regression analyses, the fundamental assumptions were verified. The normality of residuals was checked using the Shapiro–Wilk test, and homoscedasticity was assessed via scatterplots of standardized residuals against predicted values. To rule out multicollinearity, Variance Inflation Factors (VIF) were calculated, with all values remaining well below the threshold of 2.0. Regarding the k-means cluster analysis, while unsupervised learning algorithms are generally sensitive to sample size, recent extensive simulation studies demonstrate that traditional intuitions about statistical power do not fully apply to clustering. Dalmaijer et al. (2022) established that sufficient statistical power and reliable cluster recovery can be achieved with relatively small samples, recommending a minimum of N = 20 to N = 30 observations per expected subgroup, provided meaningful cluster separation exists [24]. Given our total sample size of N = 62 and the a priori theoretical assumption of two distinct subgroups (k = 2), our dataset meets this rigorous methodological threshold, yielding approximately 31 observations per cluster. Nevertheless, to maintain analytical caution, these profiles are treated as hypothesis-generating heuristics.

A significance level of *p* < 0.05 was adopted.

## 3. Results

A total of N = 62 medical professionals were included in the final analysis, all of whom provided complete questionnaire data. A purposive sampling strategy was applied, targeting personnel directly involved in the therapeutic process of oncology patients. The sample represented two key lines of care: physicians (62.9%) and nurses (37.1%).

The sociodemographic structure of the sample (Table 1) indicates a strong feminization of the group (90.3%) and a high level of education, with 87.1% holding higher education degrees. The mean age of respondents was 36.9 years (SD = 11.8), suggesting a phase of professional stabilization. In terms of worldview, Roman Catholic affiliation predominated (75.8%), alongside a notable proportion of respondents declaring themselves as non-believers (16.1%).

Verification of the reliability of the research instruments (Table 2) confirmed high internal consistency. Cronbach’s alpha coefficients for the key scales (Oncofertility Competence, Sacred Values-SVS) justified the use of advanced statistical analyses. The respondents demonstrated a moderate level of competence in oncofertility (M = 2.31), along with considerable variability in existential variables.

Table 3 presents the Pearson correlation matrix for the principal variables in the model. The analysis revealed a key psychological mechanism: religiosity is strongly associated with the value system and functions as a negative correlate of emotional acceptance of oncofertility procedures. The correlation between religiosity and emotional acceptance (r = −0.42, *p* < 0.001) was the strongest observed in the entire study. Higher levels of religiosity were associated with lower levels of emotional acceptance of oncofertility procedures, indicating a pronounced worldview conflict among religious healthcare professionals.

Religiosity also demonstrated a strong positive association with moral values (r = 0.32, *p* < 0.01), suggesting that religion constitutes a primary source of value formation within the studied group. In contrast, competence did not correlate significantly with any other variable in the bivariate analyses (r ≈ 0). Substantive knowledge was unrelated to age or religiosity. Only in the regression model did the significant role of “existential vacuum” emerge. These findings challenge the stereotype that older personnel are inherently more competent or more religiously conservative.

**Figure 1:**
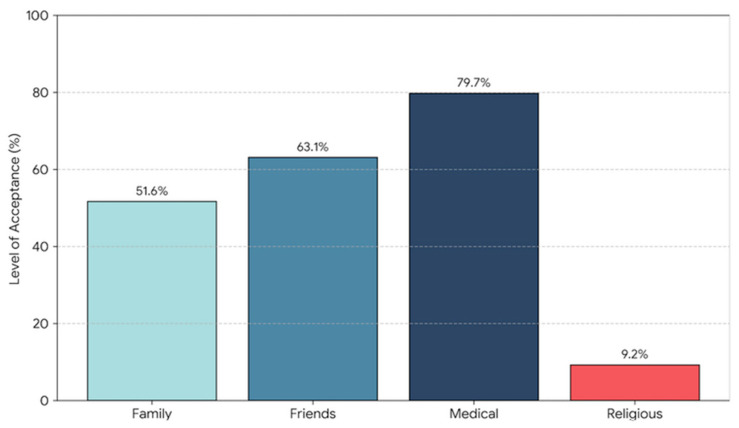
Perceived support for oncofertility procedures across reference groups.

Analysis of perceived support for oncofertility procedures (Figure 1) revealed a phenomenon of strong normative conflict within the professional environment. The medical environment was perceived as highly supportive of oncofertility procedures (M = 2.71 on a specific scale; approval index > 90%). In contrast, the religious community was perceived as strongly disapproving, exerting pressure to reject such procedures. A statistically significant divergence emerged between perceived professional mandates and religious norms regarding oncofertility. This normative incongruence suggests that religious healthcare providers may experience profound cognitive–affective dissonance, operating at the intersection of medical imperatives and personal axiological frameworks.

Despite the pilot nature of the sample (N = 62), two multiple regression models were constructed to identify potential predictors of attitudes. The model explaining competence level (adjusted R^2^ ≈ 39%) yielded novel and unexpected findings. It demonstrated that professional knowledge and experience (controlled for demographic variables) are not the sole determinants of competence. Psychological barriers play a crucial role (Table 4). The strongest negative predictor of competence was “existential vacuum”. A sense of meaninglessness significantly reduces healthcare professionals’ capacity to cope with oncofertility-related challenges, potentially hindering competence development in this domain. Rigid adherence to sacred values also exerted a suppressive effect. It is noteworthy that while competence did not exhibit significant bivariate correlations with key variables (as shown in Table 3), it emerged as significantly predicted in the multiple regression model. While this pattern might suggest a potential statistical suppression effect, it is equally likely to reflect model instability inherent to multivariate regressions conducted on small samples (N = 62). Therefore, these coefficients cannot be interpreted substantively at this stage; they represent purely mathematical associations that absolutely require independent replication before any meaningful theoretical interpretation can be drawn.

The second model (R^2^ = 23%, F(4,57) = 4.44, *p* < 0.01) explained the emotional domain (level of acceptance). Unlike competence, positive emotional attitudes were positively associated with spiritual and moral resources. Higher, internalized religiosity (β = 0.31, *p* < 0.01) exhibited a positive relationship with empathy and positive emotions. Moral values, particularly orientation toward the good of others (humanism) (β = 0.21, *p* < 0.05), correlated with greater acceptance of oncofertility procedures. Together, these factors might conceptualize what we heuristically term an ‘axiological buffer’. However, given the severe psychometric limitations of the emotional scale, this buffering mechanism cannot be treated as an empirically demonstrated effect. It remains strictly a theoretical proposition and an interpretative lens that requires robust longitudinal validation using reliable measurement tools (Table 5).

The study reveals a complex psychological mechanism underlying the functioning of personnel in pediatric oncology. The cognitive domain (competence) is threatened by deficits in meaning (“existential vacuum”), whereas the affective domain (emotions) is strongly conditioned by worldview factors (religiosity).

Cluster analysis enabled identification of two distinct employee profiles:

1. “Axiologically Integrated” individuals (approximately 37%)—characterized by high moral and religious values and low “existential vacuum”. This subgroup demonstrates higher emotional resilience and often conceptualizes their work as a mission, though they may remain susceptible to specific conscience-related moral distress.
2. “Existential Skeptics” (approximately 63%)—characterized by higher “existential vacuum” and lower perceived support. This group is most vulnerable to reduced competence, burnout, and instrumental treatment of procedures, and therefore requires the greatest level of training and institutional support.

**Figure 2:**
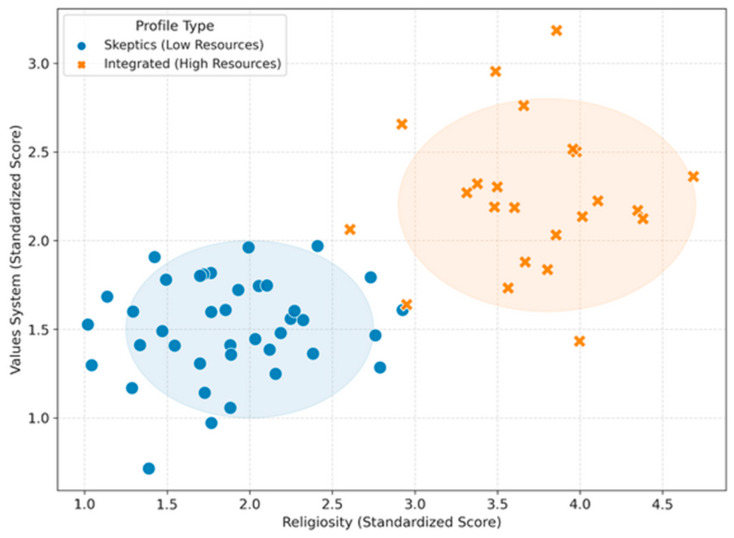
Psychological Profiles of Personnel (K-Means Clustering): scatter plot illustrating two distinct clusters based on religiosity and value systems (N = 52).

Visualization (Figure 2) confirms the presence of a dual structure within the study group—the personnel are not homogeneous but are divided into two factions with distinct psychological backgrounds.

Given the stringent constraints imposed by the sample size (N = 62), the k-means cluster analysis was operationalized exclusively as an exploratory pattern-recognition technique, rather than a definitive taxonomic classification. The decision to partition the sample into exactly two clusters (k = 2) was not arbitrary; it was determined a priori based on the dual-process theoretical framework underpinning this study, which posits a tension between clinical imperatives and personal worldview. Given the lack of a validation dataset due to the limited sample size (N = 62), traditional statistical validation indices (e.g., Silhouette scores) were considered insufficiently robust. Therefore, this k-means clustering is explicitly not presented as a definitive taxonomy of the medical population. Instead, it serves solely as an exploratory, heuristic visualization of the cognitive–affective dissociation patterns hypothesized in the regression models.

## 4. Discussion

The present study constitutes a multidimensional analysis of medical staff attitudes toward oncofertility procedures in the pediatric population. It also explores potential specific barriers to the implementation of these healthcare services. Viewed through the interpretative lens of our theoretical framework, the findings suggest that obstacles might not be exclusively systemic but could also relate to the intrapsychic structure of healthcare professionals, including their value systems, sense of meaning, and mechanisms for coping with ethical dilemmas. While these results align with international literature regarding general psychological barriers, the present study contributes a distinct perspective by identifying “existential vacuum” as a specific factor that is negatively associated with fertility preservation practices.

One of the most significant findings of the study is the absence of a correlation between age or length of professional experience and the level of competence in the field of oncofertility. This result challenges the intuitive assumption that years of clinical practice automatically translate into better preparedness for discussions concerning fertility. This observation is consistent with the findings of Tholeti et al. (2023), who demonstrated in a study of primary care physicians in India that professional seniority did not differentiate knowledge levels; as many as 60% of respondents, regardless of experience, exhibited insufficient awareness of available fertility preservation methods [9]. Similar conclusions were reported by Wang et al. (2019) in a study conducted in Shanghai among 558 reproductive healthcare professionals, where, despite generally positive attitudes, the level of detailed knowledge remained low and was not linearly associated with demographic characteristics [5]. Collectively, these findings suggest that competence in oncofertility requires active and targeted education rather than being a natural by-product of professional maturity.

A tentative contribution of the present study is the observation that “existential vacuum” (measured using the LAP-R scale) emerged in our exploratory models as a negative correlate of healthcare professionals’ communication competencies, suggesting a potential area for future empirical investigation. A diminished sense of meaning and axiological disorientation impede engagement with existentially challenging topics, such as fertility loss and the patient’s future life prospects. This finding is supported by the systematic review by Lampic and Wettergren (2019), which emphasizes that, alongside external barriers (e.g., time constraints and procedural issues), internal factors such as clinicians’ discomfort, personal beliefs, and fear of burdening patients play a crucial role [7]. This phenomenon is well illustrated by the qualitative study by Stål et al. (2025) [25], aptly titled “*Behind Closed Doors*”. The authors demonstrated that fertility-related discussions are highly non-standardized and largely dependent on clinicians’ subjective judgments (“gut feeling”), often resulting in selective provision of information [25]. The present findings suggest that such selectivity may be rooted in unresolved “existential vacuum” among healthcare professionals, leading to defensive avoidance of difficult topics.

However, a critical interrogation of these results must account for the possibility that the “existential vacuum” reported by clinicians is not the primary driver of low oncofertility competence, but rather a symptomatic reflection of systemic neglect. In highly bureaucratic healthcare systems, the perceived meaninglessness may be a form of ‘learned helplessness’ resulting from a lack of clear protocols and institutional support. While our study emphasizes intrapsychic factors, it is plausible that in different organizational cultures—where roles are strictly defined—individual existential attitudes might play a less prominent role than observed in our Polish cohort. This alternative perspective suggests that the interaction between personality constructs and organizational climate requires further investigation to avoid psychological over-determinism.

A major systemic problem widely described in the literature is the transfer of responsibility for fertility-related discussions to other members of the healthcare team. Keim-Malpass et al. (2018), in a study of oncology nurses, highlighted a paradox whereby nurses perceived fertility education as highly important while simultaneously viewing it as outside their professional scope, resulting in 76.9% of respondents rarely or never providing such information [26]. This issue is also embedded in the broader context of supportive care implementation. Mittal et al. (2022), analyzing barriers to recruitment in supportive care clinical trials for adolescents and young adults (AYA), identified low prioritization and the absence of dedicated leaders (“champions”) as key obstacles [16]. The present study adds that this phenomenon may stem not only from time constraints but also from an internal normative conflict, whereby fertility-related issues are subconsciously devalued in the face of life-saving imperatives (the so-called medical imperative).

The negative correlation identified in the present study between centrality of religiosity and emotional acceptance of oncofertility procedures points to the presence of an internal normative conflict. Healthcare professionals appear to be situated between the expectations of the professional medical environment, which promotes these procedures, and religious communities perceived as opposing them. This issue is less frequently addressed in Western literature, which tends to focus primarily on logistical barriers. However, Wang et al. (2019) also suggest that cultural and traditional determinants may shape perceptions of fertility, particularly in societies characterized by strong conservative norms, such as Poland [5].

In contrast to our emphasis on psychological determinants, some studies in larger, multi-center international cohorts have highlighted that structural barriers, such as the lack of specialized oncofertility coordinators or high costs of procedures, remain the primary obstacles to implementation. Our findings do not invalidate these systemic factors but rather suggest that in settings where systemic issues are constant, psychological and axiological variables may act as secondary filters that further differentiate clinicians’ engagement.

The findings of the present study have important implications for the design of educational interventions. Paynter et al. (2026), in a study of healthcare professionals from Australia and New Zealand, emphasized the need for formal procedural training, pointing to deficits in technical and systemic competencies [27]. Similarly, Kemertzis et al. (2018) demonstrated that implementation of a *Fertility Preservation Toolkit* increased healthcare professionals’ self-confidence from 40% to 70% [28]. However, the present data indicate that the provision of tools or procedural knowledge alone may be insufficient if the existential level is not adequately addressed. To overcome the barriers related to “existential vacuum” and value conflicts, educational programs should incorporate a reflective component that provides space for discussion of personal emotions, ethical dilemmas, and the meaning of fertility in human life. Without such an approach, as demonstrated by the findings of this study, theoretical knowledge is unlikely to translate into standardized clinical practice.

## 5. Study Limitations

Despite providing novel insights into the intrapsychic and existential determinants of medical staff attitudes toward pediatric oncofertility, this study has several methodological limitations that must be carefully considered when interpreting the findings.

First, while the absolute sample size (N = 62, including 39 physicians) may appear modest, it must be evaluated within the context of the national healthcare system. With only approximately 180 pediatric oncologists currently practicing in Poland, our physician sample successfully captures nearly 22% of the entire national target population. While the purposive sampling strategy carries an inherent risk of selection bias, capturing such a substantial fraction of this highly specialized, hard-to-reach workforce provides a robust foundation for these preliminary findings.

Second, the cross-sectional design of the study precludes the establishment of causal relationships between “existential vacuum”, religiosity, and professional competence. It remains to be determined in future longitudinal studies whether an “existential vacuum” is a primary cause of reduced competence, or rather a secondary consequence of chronic moral distress and professional burnout typical in pediatric oncology. Additionally, reliance on self-report measures introduces the risk of social desirability bias, particularly when participants are asked to report on sensitive ethical dilemmas and subjective professional competencies.

Third, from a statistical perspective, the limited sample size restricts the power of the advanced analytical models. The multiple regression and k-means cluster analyses presented in this study—while offering a valuable conceptual framework, such as the identification of the “Axiologically Integrated” and “Existential Skeptics” profiles—should be interpreted as strictly exploratory and illustrative. They require replication on a larger, representative scale to establish definitive professional typologies.

Finally, a critical psychometric limitation is the very low internal consistency of the emotional acceptance scale (Cronbach’s α = 0.268). Although this resulted from the use of a shortened, one-dimensional tool designed specifically to minimize respondent burden in high-stress clinical settings, it indicates substantial heterogeneity in how the affective domain was measured. Therefore, findings pertaining to emotional acceptance must be interpreted with extreme caution and viewed strictly as a preliminary signal that necessitates rigorous re-evaluation using standardized, multi-item psychometric instruments in future research. We explicitly acknowledge that our current findings regarding the affective domain are statistically fragile, and applying different measurement strategies would likely result in different structural relationships.

Despite these limitations, this preliminary report sheds necessary light on an under-researched area of psychological barriers in oncofertility, providing a foundational starting point for further large-scale analyses.

## 6. Practical Implications and Directions for Future Research

Using existential emptiness and normative conflict as interpretative lenses, the results of this exploratory study generate the hypothesis that implementation strategies might benefit from incorporating existential dimensions. Traditional educational models based solely on the transfer of factual knowledge prove insufficient in the face of intrapsychic barriers.

Given the lack of correlation between length of professional experience and competence, training programs must go beyond procedural instruction. The findings tentatively support the inclusion of modules addressing bioethical dilemmas and values clarification, which may enable healthcare professionals to reduce cognitive dissonance. 

To tentatively mitigate potential bias stemming from individual beliefs, future intervention designs might consider exploring the utility of standardized decision-support tools.

The phenomenon of diffusion of responsibility and the perception of oncofertility as a task “beyond one’s professional scope” calls for organizational changes. The establishment of dedicated oncofertility teams within healthcare centers could be hypothesized as a potential organizational improvement, though empirical validation of its efficacy is needed.

A shift from cross-sectional research designs to longitudinal models is necessary to determine whether existential emptiness constitutes a primary cause of low competence or a secondary consequence of chronic moral distress and professional burnout in oncology. Furthermore, it is essential to examine the extent to which the strong normative conflict identified in Poland (medicine versus religion) is specific to religiously homogeneous cultures. Comparing these findings with data from Asian contexts or secularized Western societies would allow for the differentiation of universal psychological barriers from locally conditioned factors. Future analyses should also investigate the predictive value of healthcare professionals’ level of existential maturity on distal patient outcomes, such as decision regret and post-treatment quality of life, enabling a comprehensive assessment of the clinical significance of the phenomena under study.

## 7. Conclusions

The study challenges the widespread belief in a linear relationship between length of professional experience and preparedness for fertility-related counseling, questioning the assumption that readiness to discuss fertility is automatically acquired through general clinical experience.

Viewed through our proposed interpretative framework, the exploratory data suggest that deficits in a sense of meaning might potentially be associated with a reduced readiness to engage in challenging conversations about procreation with pediatric patients. The data tentatively suggest that a lack of perceived meaning may hinder communication with patients alongside, or potentially independently of, established systemic barriers. However, critically interrogating these findings requires acknowledging alternative theoretical frameworks. Given the cross-sectional nature of this study, causality cannot be definitively established. It is entirely plausible that the observed relationship operates in reverse: rather than being a primary intrapsychic barrier that inhibits clinical competence, “existential vacuum” might be a secondary symptom or consequence of chronic moral distress and professional burnout, which are highly prevalent in pediatric oncology. Continuous exposure to suffering and unresolved systemic tensions may deplete healthcare providers’ axiological resources over time. Future longitudinal research is required to disambiguate the directionality of this mechanism.

The findings heuristically point toward a potential cognitive dissonance between the medical imperative and the worldview of healthcare professionals within the examined cultural context, though this requires further validation.

Consequently, our exploratory data tentatively suggest that future systemic interventions might benefit from evaluating the introduction of external decision-support tools, aiming to standardize fertility preservation processes regardless of subjective beliefs.

Consequently, these preliminary observations generate the hypothesis that the implementation of oncofertility standards might benefit from educational models that incorporate reflective practice, aiming to address potential moral distress and axiological dilemmas.

## Figures and Tables

**Table 1:** Sociodemographic characteristics of the study group (N = 62).

Variable	Category	N	%
Professional Group	Physicians	39	62.9
	Nurses	23	37.1
Gender	Female	56	90.3
	Male	6	9.7
Education	Higher education	54	87.1
	Secondary education	8	12.9
Religious affiliation	Roman Catholic	47	75.8
	Non-believers	10	16.1
	Other	5	8.1

**Table 2:** Descriptive statistics and reliability of variables included in the model (N = 62).

Variable	M	SD	Me	Min–Max	Cronbach’s α
Oncofertility Competence (WO_SCORE)	2.31	0.56	2.33	1.07–4.00	0.865
“Existential Vacuum” (LAP_EV)	2.20	0.72	2.00	1.00–4.00	0.790
Sacred Values (SVS_SAC)	1.70	0.44	1.71	0.57–2.00	0.932
Emotions toward procedures	3.12	0.68	3.20	1.00–5.00	0.268 *

* Crucially, the emotional acceptance subscale exhibited severe internal consistency deficits (Cronbach’s α = 0.268). However, it is imperative to conceptualize this measure not as a traditional reflective scale (where items are expected to be highly intercorrelated), but rather as a formative index of diverse, often ambivalent emotional reactions to complex clinical scenarios. This substantial psychometric limitation likely arises from the utilization of a truncated, unidimensional item set. Consequently, statistical inferences regarding the affective domain must be interpreted strictly as highly tentative heuristic signals. Treating this index as a dependent variable in regression models poses a risk of model instability, and it is highly probable that alternative, standardized measurement strategies would yield different affective patterns.

**Table 3:** Pearson correlation matrix for psychological and professional variables (N = 65).

Variable	Age	Religiosity (C-15)	Moral Values (SVS)	Life Purpose (LAP)	Competence (WO)	Emotional Acceptance
Age	-	0.00	−0.08	0.04	−0.00	0.19
Religiosity (C-15)	0.00	-	0.32 **	0.14	−0.00	−0.42 ***
Moral Values (SVS)	−0.08	0.32 **	-	0.12	−0.15	−0.25 *
Life Purpose (LAP)	0.04	0.14	0.12	-	0.09	−0.13
Competence (WO)	−0.00	−0.00	−0.15	0.09	-	−0.07
Emotional Acceptance	0.19	−0.42 ***	−0.25 *	−0.13	−0.07	-

Significance levels: * *p* < 0.05; ** *p* < 0.01; *** *p* < 0.001.

**Table 4:** Regression analysis results for the dependent variable: Competence.

Predictor	B	SE	β	t	*p*
Intercept	3.15	0.42	-	7.50	<0.001
“Existential Vacuum”	−0.34	0.10	−0.42	−3.32	0.001
Sacred Values	−0.28	0.11	−0.31	−2.54	0.013
Age	−0.01	0.01	−0.15	−1.12	0.268

**Table 5:** Multiple regression model for the dependent variable: Positive emotional attitude toward oncofertility.

Predictor	β	t	*p*	95% CI
Religiosity (C-15)	0.31	2.65	<0.01	[0.08, 0.54]
Moral Values (SVS)	0.21	2.12	<0.05	[0.02, 0.40]
Purpose Seeking (LAP) ^1^	0.16	1.98	<0.05	[0.00, 0.32]
Age	0.05	0.45	0.654	[−0.15, 0.25]

^1^ The “Purpose Seeking” variable was included as a complementary personal resource, in accordance with correlation analysis results.

## Data Availability

Data sharing is not applicable. The data contain sensitive information regarding the religious beliefs and value systems of the personnel; public disclosure could potentially affect the reputation of the center based on personal beliefs rather than professional merit.

## Abbreviations

The following abbreviations are used in this manuscript:

SVS	Scheler Value Scale
LAP-R	Life Attitude Profile—Revised
CRS-15	Centrality of Religiosity Scale
GCP	Good Clinical Practice
SD	Standard deviation
M	Mean
LAP	Life Attitude Profile
AYA	Adolescents and young adults
VIF	Variance Inflation Factors
Me	Median
